# Supplementary material for: Timely vaccine strain selection and genomic surveillance improves evolutionary forecast accuracy of seasonal influenza A/H3N2
Source: medRxiv. 2024 Sep 13:2024.09.11.24313489. Preprint. [Version 1] doi: 10.1101/2024.09.11.24313489 (PMC11419249; doi:10.1101/2024.09.11.24313489)
Supplement: Supplement 1 [file NIHPP2024.09.11.24313489v1-supplement-1.pdf]

medRxiv preprint doi: https://doi.org/10.1101/2024.09.11.24313489; this version posted September 13, 2024. The copyright holder for this preprint (which was not certified by peer review) is the author/funder, who has granted medRxiv a license to display the preprint in perpetuity.

It is made available under a CC-BY 4.0 International license .

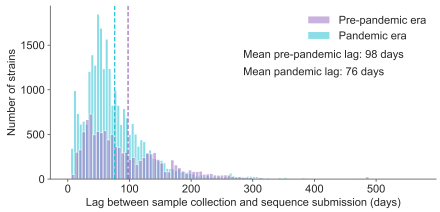

Figure 1—figure supplement 1. Distribution of submission lags in days for the pre-pandemic era (2019-2020 in blue) and pandemic era (2022-2023 in orange). Vertical dashed lines represent mean lags for each distribution.

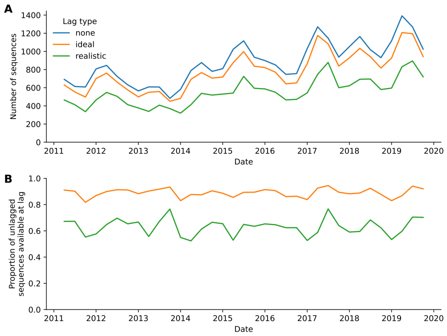

Figure 1—figure supplement 2. A) Number of A/H3N2 sequences available per timepoint and lag type. B) Proportion of all A/H3N2 sequences without lag per timepoint and lag type.

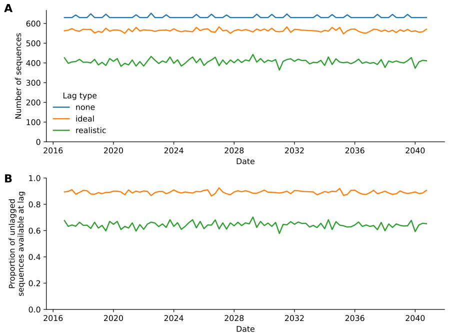

Figure 1—figure supplement 3. A) Number of simulated A/H3N2-like sequences available per timepoint and lag type. B) Proportion of all simulated A/H3N2-like sequences without lag per timepoint and lag type.

medRxiv preprint doi: https://doi.org/10.1101/2024.09.11.24313489; this version posted September 13, 2024. The copyright holder for this preprint (which was not certified by peer review) is the author/funder, who has granted medRxiv a license to display the preprint in perpetuity.

It is made available under a CC-BY 4.0 International license .

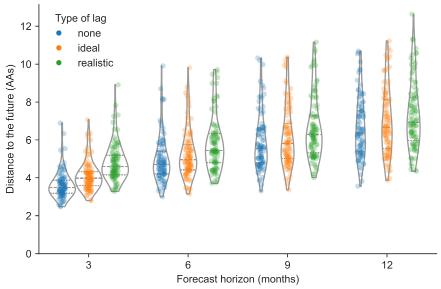

Figure 2—figure supplement 1. Distance to the future per timepoint (AAs) for simulated A/H3N2-like populations by forecast horizon and submission lag type based on forecasts from the "true fitness" model.

Figure 2—figure supplement 1—source data 1. Distances to the future for simulated A/H3N2-like populations; see https://zenodo.org/records/13742375

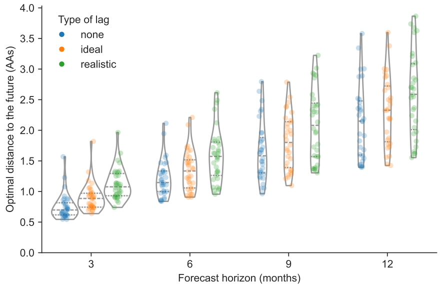

Figure 2—figure supplement 2. Optimal distance to the future per timepoint (AAs) for natural A/H3N2 populations by forecast horizon and submission lag type based on posthoc empirical fitness of the initial population.

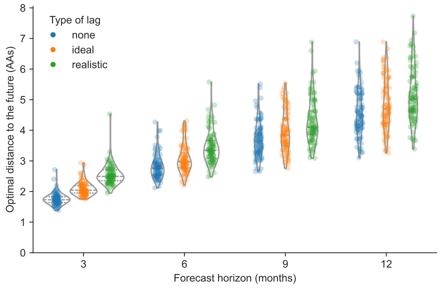

Figure 2—figure supplement 3. Optimal distance to the future per timepoint (AAs) for simulated A/H3N2-like populations by forecast horizon and submission lag type based on posthoc empirical fitness of the initial population.

medRxiv preprint doi: https://doi.org/10.1101/2024.09.11.24313489; this version posted September 13, 2024. The copyright holder for this preprint (which was not certified by peer review) is the author/funder, who has granted medRxiv a license to display the preprint in perpetuity.

It is made available under a CC-BY 4.0 International license .

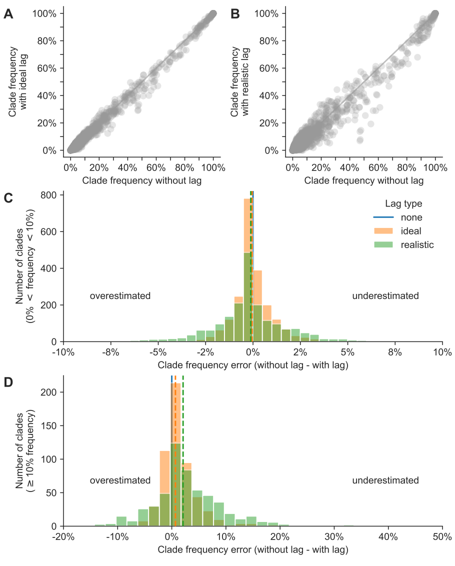

Figure 3—figure supplement 1. Clade frequency errors between simulated A/H3N2-like HA populations with ideal or realistic submission lags and populations without any submission lag.

Figure 3—figure supplement 1—source data 1. Current and future clade frequencies for simulated A/H3N2-like populations by forecast horizon and submission lag type; see https://zenodo.org/records/13742375

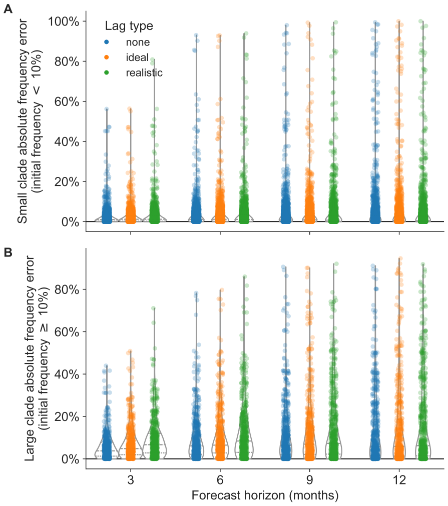

Figure 4—figure supplement 1. Absolute forecast clade frequency errors for simulated A/H3N2-like HA populations by forecast horizon in months and submission lag type (none, ideal, or realistic) for A) small clades (<10% initial frequency) and B) large clades (≥10% initial frequency).

medRxiv preprint doi: https://doi.org/10.1101/2024.09.11.24313489; this version posted September 13, 2024. The copyright holder for this preprint (which was not certified by peer review) is the author/funder, who has granted medRxiv a license to display the preprint in perpetuity.

It is made available under a CC-BY 4.0 International license .

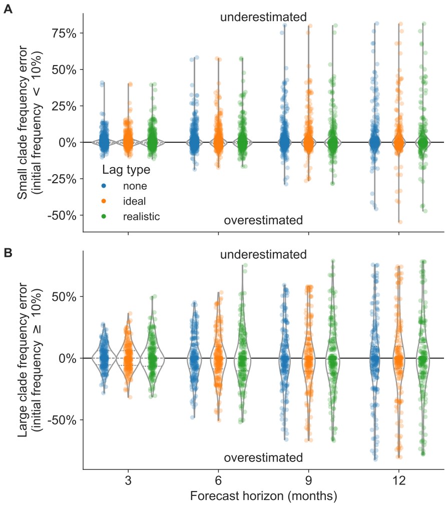

Figure 4—figure supplement 2. Forecast clade frequency errors for natural A/H3N2 HA populations by forecast horizon in months and submission lag type (none, ideal, or realistic) for A) small clades (<10% initial frequency) and B) large clades (≥10% initial frequency).

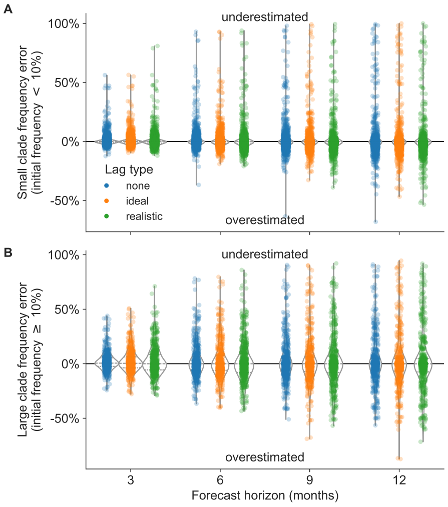

Figure 4—figure supplement 3. Forecast clade frequency errors for simulated A/H3N2-like HA populations by forecast horizon in months and submission lag type (none, ideal, or realistic) for A) small clades (<10% initial frequency) and B) large clades (≥10% initial frequency).

medRxiv preprint doi: https://doi.org/10.1101/2024.09.11.24313489; this version posted September 13, 2024. The copyright holder for this preprint (which was not certified by peer review) is the author/funder, who has granted medRxiv a license to display the preprint in perpetuity.

It is made available under a CC-BY 4.0 International license .

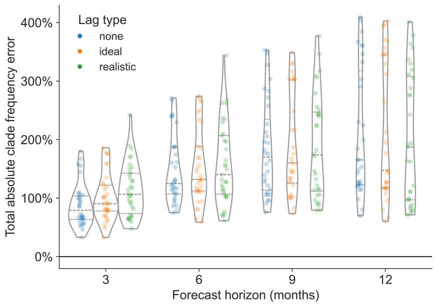

Figure 5—figure supplement 1. Distribution of total absolute clade frequency errors summed across clades per future timepoint for A/H3N2 populations. We calculated the effects of interventions as the difference between these values per future timepoint under the status quo (12-month forecast horizon and realistic submission lag) and specific interventions.

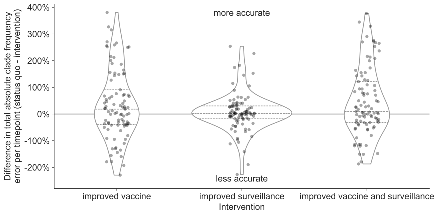

Figure 5—figure supplement 2. Improvement of clade frequency errors for simulated A/H3N2-like populations between the status quo and realistic interventions.

Figure 5—figure supplement 2—source data 1. Differences in total absolute clade frequency error per future timepoint and clade between the status quo and realistic interventions for simulated A/H3N2-like populations; see https://zenodo.org/records/13742375

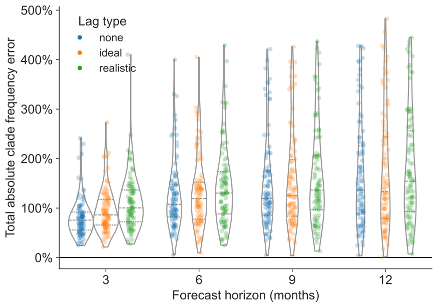

Figure 5—figure supplement 3. Distribution of total absolute clade frequency errors summed across clades per future timepoint for simulated A/H3N2-like populations.

medRxiv preprint doi: https://doi.org/10.1101/2024.09.11.24313489; this version posted September 13, 2024. The copyright holder for this preprint (which was not certified by peer review) is the author/funder, who has granted medRxiv a license to display the preprint in perpetuity.

It is made available under a CC-BY 4.0 International license .

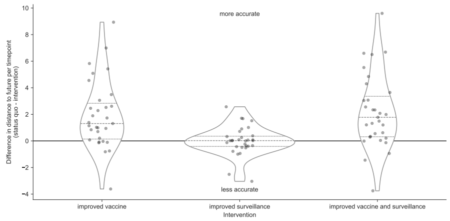

Figure 5—figure supplement 4. Improvement of distances to the future (AAs) for A/H3N2 populations between the status quo (12-month forecast horizon and realistic submission lags) and realistic interventions. The effects of interventions are the differences between distances to the future per future timepoint under the status quo and specific interventions.

Figure 5—figure supplement 4—source data 1. Improvement of distances to the future per future timepoint for A/H3N2 populations; see https://zenodo.org/records/13742375

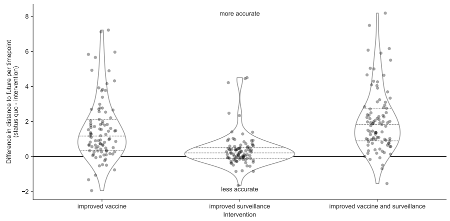

Figure 5—figure supplement 5. Improvement of distances to the future (AAs) for simulated A/H3N2-like populations between the status quo (12-month forecast horizon and realistic submission lags) and realistic interventions. The effects of interventions are the differences between distances to the future per future timepoint under the status quo and specific interventions.

Figure 5—figure supplement 5—source data 1. Improvement of distances to the future per future timepoint for simulated A/H3N2-like populations; see https://zenodo.org/records/13742375

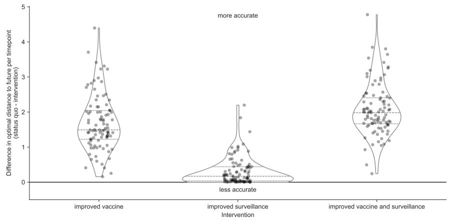

Figure 6—figure supplement 1. Improvement of optimal distances to the future (AAs) for simulated A/H3N2-like populations between the status quo (12-month forecast horizon and realistic submission lags) and realistic interventions.

Figure 6—figure supplement 1—source data 1. Improvement of optimal distances to the future per future timepoint for simulated A/H3N2-like populations; see https://zenodo.org/records/13742375
